# Editorial: Immunological Tolerance in Transplantation: More Than Deletion

**DOI:** 10.3389/fimmu.2022.959115

**Published:** 2022-06-30

**Authors:** Nina Pilat, Thomas Wekerle, Edward K. Geissler, Jonathan Sprent

**Affiliations:** ^1^ Division of Transplantation, Department of General Surgery, Medical University of Vienna, Vienna, Austria; ^2^ University Hospital Regensburg, Department of Surgery, University of Regensburg, Regensburg, Germany; ^3^ Immunology Division, Garvan Institute of Medical Research, Darlinghurst, NSW, Australia; ^4^ St Vincent's Clinical School, University of New South Wales, Sydney, NSW, Australia

**Keywords:** immunological tolerance, transplantation, mixed chimerism, Tregs, tolerance approaches, intragraft tolerance

## Introduction

In transplantation medicine, induction of donor-specific tolerance remains the “holy grail” to protect organs from rejection. Indeed, clinicians and immunologists still struggle to prevent rejection and improve long-term graft survival, with an acceptable safety profile. Transplant recipients still require life-long chronic immunosuppression, which results in drug-specific side effects and complications from nonspecific suppression of the immune response.

Tolerance is presumed to be an active equilibrium between allo/auto-reactive and regulatory immune mechanisms. Antigen-specific T regulatory cells (Tregs) as well as other regulatory cell subsets are critical for the maintenance of self-tolerance and have been recognized as promising and potent therapeutic tools in transplantation. However, although numerous tolerance approaches have been developed in preclinical animal studies (mostly rodents), translation into large animal models or clinical application has almost always failed the ultimate test.

This Research Topic brings together 9 articles that aim to tackle the most important questions about tolerance mechanisms and their translation into the clinic.

## Article Collection

The mixed chimerism approach, which involves co-transplantation of hematopoietic stem cells (HSCT) and a solid organ from the same donor, is the only tolerance approach which has been successfully translated into a clinical setting. However, widespread clinical application of this approach is still impeded by the often severe effects of cytotoxic recipient pretreatment and the risk of graft-versus-host disease (GVHD). To this point, Fehr et al recently reported on the first patients in Europe receiving combined kidney and HSCT for successful tolerance induction. Three patients receiving kidney and HSCT from an HLA-identical sibling donor were successfully weaned from immunosuppression without rejection or GVHD episodes. At the time of this writing, these three patients have been off immunosuppression for 4 years, 19 and 8 months. Importantly, all three patients showed excellent responses to vaccinations against SARS-CoV-2 in the ongoing pandemic with strong humoral and cellular specific immunity. The protocol used was previously developed by the Stanford group, who have decades of preclinical and clinical experience with the chimerism tolerance approach in HLA-identical siblings. Lowsky and Strober have contributed an excellent review on this topic, pointing out differences in the mechanisms of organ graft acceptance in mixed chimeras versus full chimeras.

Another excellent review about tolerance mechanisms induced by HSCT was contributed by Podesta and Sykes. Whereas tolerance is mainly maintained by central deletion of graft-reactive donor T cells in full chimeras, tolerance mechanisms in transient mixed chimeras rely largely on Treg-mediated suppression plus peripheral (intragraft) deletion of donor-reactive recipient T-cell clones. Although these mechanisms are not fully understood, a better understanding of intragraft tolerance mediated by regulatory mechanisms will be important for devising new approaches for preventing chronic rejection and allo-sensitization, and eventually achieving the ultimate goal of “one transplant for life”. Currently, however, universally accepted and validated biomarkers for clearly defining tolerance in transplant patients have remained elusive. Here, the authors summarized the most important mechanistic studies on tolerance induction as well as cutting-edge methods to identify patients worth considering for safe immunosuppression withdrawal.

In another comprehensive review by Hall et al (Clonal deletion and T cell mechanisms that mediate transplant tolerance), the authors sum up historical aspects that have led to development of the clonal deletion theory before the discovery of T cells, and an improved understanding of the immune responses. Furthermore, models of operational tolerance, the concept of split tolerance, and the importance of regulatory mechanisms are summarized. Here, the authors discuss the role of clonal deletion in transplant tolerance in light of regulatory T cells and their ability to mediate antigen-specific tolerance.

With regard to inducing tolerance, extracorporal photopheresis (ECP) is an apheresis procedure involving the removal of peripherally circulating white blood cells, addition of a light sensitizer, exposure to UV light, and return of the cells to the patient. By mechanisms that are yet not fully understood, ECP can promote the transition from an inflammatory state to a pro-tolerance state in some settings. Originally approved for cutaneous T cell lymphoma, ECP is used for prevention/treatment of GVHD after HCST, but also to reduce rejection in solid organ transplantation. Dieterlen et al reported on a novel immune monitoring assay for ECP treatment after heart transplantation. Based on flow cytometric measurements of dendritic cell subsets and Tregs, the authors proposed classification criteria to identify patient-specific immunological improvement. This tool may allow optimization of ECP treatment duration in heart transplant patients; however, a multicenter study would be needed for validation of clinical use.

Solid organ and cellular grafts contain donor-derived bone marrow-derived hematopoietic cells. The role of these so-called “passenger leukocytes” in transplantation immunology is complex. Thus, although it has long been known that these cells can initiate graft rejection, in certain settings they can also contribute to graft acceptance. In this respect, Hitz et al aimed to characterize the expression of killer cell immunoglobulin-like receptors (KIR) on donor and recipient myeloid cells in recipient blood in double-lung transplant patients. For these patients, the authors report enhanced frequencies of donor NK and T cells expressing regulatory KIR; in addition, they find evidence suggestive of pre-activation of donor cells during the ischemic phase. In view of these findings, donor NK and T cells in lung transplant recipients may both play a role in regulating alloresponses and contributing to allograft tolerance.

In a murine model of Treg-mediated skin graft survival, Steiner et al investigated the mechanisms by which graft-resident leucocytes impact skin allograft survival. In this model, diminution of graft-resident leucocytes leads to a decreased infiltration of recipient T cells into the graft, thereby switching the intragraft cell composition to a more tolerogenic milieu. This study demonstrates the importance of donor-derived leucocytes in recipient sensitization and allograft rejection, and suggests that long-term graft survival could be improved by targeting the early stages of T cell allorecognition.

With regard to initial stem cell engraftment, Bhat et al aimed to evaluate immune reconstitution following haplo-HSCT for treatment of sickle cell disease. The authors performed longitudinal flow cytometric analysis of leucocyte subsets in PBMCs and cytokine analysis in serum of patients post non-myeloablative haplo-HSCT. Their data suggest that successful HSCT engraftment may depend upon an early increase in numbers of myeloid-derived suppressor cells. Notably, successful engraftment correlated with elevated levels of suppressive cytokines. Hence, the latter could serve as potential prognostic markers in predicting successful engraftment after HSCT.

Endoribonuclease Regnase-1 is expressed in T and B cells and is known to be an important feedback mechanism for negative regulation of immune responses. Here, Kong et al developed a transgenic mouse model expressing mutant Regnase-1 with improved *in vivo* stability. These transgenic mice are lymphopenic and show a marked decrease in numbers of mature T cells, leading to a failure to reject fully MHC-mismatched skin grafts. The authors demonstrated that the paucity of T cells reflected impaired T cell development in the thymus due to disrupted TCR signaling during positive selection. This study suggests Regnase-1 as a therapeutic target for the modulation of T cell function.

## Conclusion

This Research Topic highlights recent preclinical and clinical findings on allotransplantation and provides new insights on the complex immunoregulatory mechanisms that induce and impede immunological tolerance towards allografts. Further work on this topic may lead eventually to the realization of the ultimate goal of achieving permanent graft survival without the need for immunosuppression.

## Author Contributions

All authors listed have made a substantial, direct, and intellectual contribution to the work and approved it for publication.

## Conflict of Interest

The authors declare that the research was conducted in the absence of any commercial or financial relationships that could be construed as a potential conflict of interest.

## Publisher’s Note

All claims expressed in this article are solely those of the authors and do not necessarily represent those of their affiliated organizations, or those of the publisher, the editors and the reviewers. Any product that may be evaluated in this article, or claim that may be made by its manufacturer, is not guaranteed or endorsed by the publisher.

